# Identification and Counting of Pirapitinga *Piaractus brachypomus* Fingerlings Fish Using Machine Learning

**DOI:** 10.3390/ani14202999

**Published:** 2024-10-17

**Authors:** Alene Santos Souza, Adriano Carvalho Costa, Heyde Francielle do Carmo França, Joel Jorge Nuvunga, Gidélia Araújo Ferreira de Melo, Lessandro do Carmo Lima, Vitória de Vasconcelos Kretschmer, Débora Ázara de Oliveira, Liege Dauny Horn, Isabel Rodrigues de Rezende, Marília Parreira Fernandes, Rafael Vilhena Reis Neto, Rilke Tadeu Fonseca de Freitas, Rodrigo Fortunato de Oliveira, Pedro Henrique Viadanna, Brenno Muller Vitorino, Cibele Silva Minafra

**Affiliations:** 1 Department of Science Animal, Federal Institute of Education, Science and Technology of Goiás (IF Goiano), Campus Rio Verde, Goiana South Highway, Km 01, Rio Verde 75901-970, GO, Brazil; alenesantos47@gmail.com (A.S.S.); heyde.franca@ifgoiano.edu.br (H.F.d.C.F.); gideliarv.melo@gmail.com (G.A.F.d.M.); lessandro_lima@hotmail.com (L.d.C.L.); vitoria.vasconcelos@estudante.ifgoiano.edu.br (V.d.V.K.); deboraazara@gmail.com (D.Á.d.O.); liegedouny@gmail.com (L.D.H.); isabel.r.rezende@gmail.com (I.R.d.R.); lilinhaparreira@gmail.com (M.P.F.); fortunatorodrigo@ymail.com (R.F.d.O.); brenno.muller@estudante.ifgoiano.edu.br (B.M.V.); cibele.minafra@ifgoiano.edu.br (C.S.M.); 2 Center of Excellence in Agri-Food Systems and Nutrition, Eduardo Mondlane University, Julius Nyerere, n° 3453, Maputo P.O. Box 257, Mozambique; 3 Faculty of Science and Technology, Joaquim Chissano University, Grande Maputo Street, 88, Maputo P.O. Box 1110, Mozambique; 4 Department of Science Animal, State University Paulista Júlio de Mesquita Filho, Nelson Brihi Badur, 480, Registro 11900-000, SP, Brazil; rafael.vilhena@unesp.br; 5 Department of Science Animal, Federal University of Lavras, Ignácio Valentin, Lavras 37200-900, MG, Brazil; rilke@ufla.br; 6 Department of Biological Sciences, College of Arts and Sciences, Washington State University, Pullman, WA 99163, USA; pedroh1986@gmail.com

**Keywords:** aquaculture, automation, detection algorithm, neural network, South American round fish

## Abstract

Identifying and counting fish are crucial for managing stocking, harvesting, and marketing of farmed fish. Researchers have used convolutional networks for these tasks and explored various approaches to enhance network learning. Batch normalization is one technique that improves network stability and accuracy. This study aimed to evaluate machine learning for identifying and counting pirapitinga *Piaractus brachypomus* fry with different batch sizes. The researchers used one thousand photographic images of Pirapitinga fingerlings, labeled with bounding boxes. They trained the adapted convolutional network model with batch normalization layers added at the end of each convolution block. They set the training to one hundred and fifty epochs and tested batch sizes of 5, 10, and 20. Furthermore, they measured network performance using precision, recall, and mAP@0.5. Models with smaller batch sizes performed less effectively. The training with a batch size of 20 achieved the best performance, with a precision of 96.74%, recall of 95.48%, mAP@0.5 of 97.08%, and accuracy of 98%. This indicates that larger batch sizes improve accuracy in detecting and counting pirapitinga fry across different fish densities.

## Simple Summary

Identification and counting of fish are relevant tools for managing the stocking, harvesting, and marketing of farmed fish. Researchers have used convolutional networks for these purposes and employed various approaches to improve network learning. Batch normalization is one technique that enhances network stability and accuracy. The study aimed to evaluate machine learning for identifying and counting pirapitinga *Piaractus brachypomus* fry with different batch sizes. The researchers used one thousand photographic images of Pirapitinga fingerlings, labeled with bounding boxes. They trained the adapted convolutional network model with batch normalization layers inserted at the end of each convolution block. The training involved one hundred and fifty epochs, with batch sizes for normalization set to 5, 10, and 20.

## 1. Introduction

Identifying and counting fish are relevant tools for managing the stocking, harvesting, and marketing of farmed fish. Researchers have developed several studies to facilitate management and automate fish counting using deep learning with convolutional neural networks (CNNs) [1,2,3]. These tools automate management by reducing or eliminating animal handling, making the process easier, faster, and more accurate than manual methods [4,5].

Manual counting methods involve sampling individuals in containers according to species and their average weight. In commercial fish fry operations, practitioners commonly use estimation methods involving sieves. Alternatively, some techniques involve counting each fry individually. However, these methods have notable drawbacks, including stress and secondary infections in the animals and increased strain on labor, which can lead to errors and reduced accuracy in counting [6].

In aquaculture systems, automating the counting process is less invasive and more effective and accurate than manual methods. Convolutional neural networks offer a promising solution for developing automatic counters, as they can detect and identify fish from images and videos. This approach speeds up handling and reduces the stress and injuries associated with manual counting of animals [7].

Convolutional neural networks, combined with detection algorithms, offer enhanced robustness, speed, and lower computational costs for tasks involving fish recognition, classification, and detection [8,9]. Reports indicate that detecting fish in underwater environments achieved a precision of 95.7% and an average precision (mAP@0.5) of 95.4% using the Yolov5 detection algorithm [6]. These results demonstrate the effectiveness and performance of detection algorithms, allowing for more accurate identification of aquatic animals in complex environments [10,11]. Despite these advantages, challenges remain in learning these algorithms, including issues such as fish occlusions due to high densities, low image quality, and varying lighting conditions.

Researchers have proposed various approaches to address these challenges and enhance the performance and robustness of detection algorithms during training [8,12,13]. One such approach is using the batch normalization layer, which improves network stability and provides greater accuracy and learning performance. Additionally, this technique contributes to model regularization, reducing the risk of overfitting [14]. The optimal batch size varies depending on the task, the specific problem, and available computational resources.

Research on the detection and counting of fish fingerlings is limited. Most studies focus on identifying and recognizing larger underwater marine species in images and videos. Therefore, research that examines normalization and batch size for detecting commercial fingerling species is particularly valuable. Fingerlings from the Serrasalmidae family, which includes economically important South American fish such as pacu, tambaqui, and pirapitinga, as well as their hybrids, play a significant role in Latin America [15,16,17]. Our objective was to evaluate the impact of batch size normalization on the counting of Pirapitinga (*Piaractus brachypomus*) fingerlings using digital photographic images.

## 2. Materials and Methods

### 2.1. Dataset

**Figure 1:**
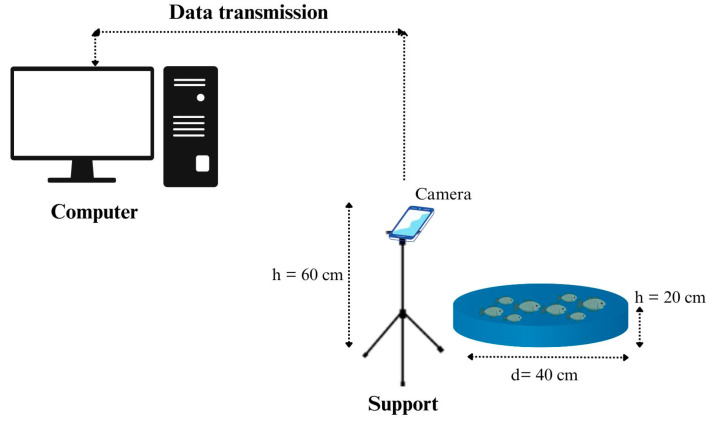
Collection platform scheme. (h) Height in centimeter (cm); (d) Diameter in cm.

**Figure 2:**
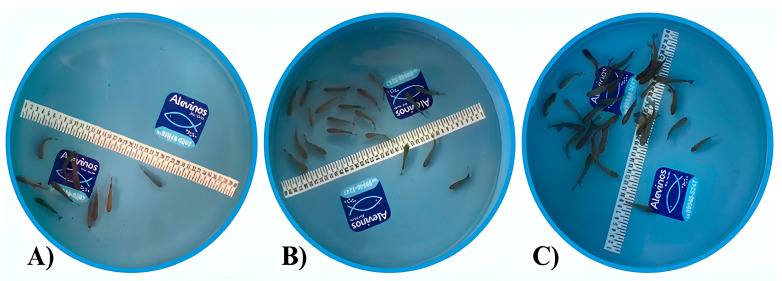
Different densities of pirapitinga fingerlings were used in the study. (**A**) Tank with 10 fingerlings; (**B**) Tank with 20 fingerlings; and (**C**) Tank with 30 fingerlings.

We collected the dataset for the study at Alevinos Rio Verde in Goiás, Brazil, where the company provided the space and pirapitinga specimens for image collection. We took one thousand images of pirapitinga fingerlings (average length 3.5 cm) from a blue-bottomed tank with a capacity of 25 L and a diameter of 40 cm (Figure 1). We stocked the fish at densities ranging from 10 to 50 fingerlings (Figure 2). We used an iPhone XR with a 12-megapixel camera and a resolution of 4608 × 2592 to capture the images.

### 2.2. Dataset Labeling

**Figure 3:**
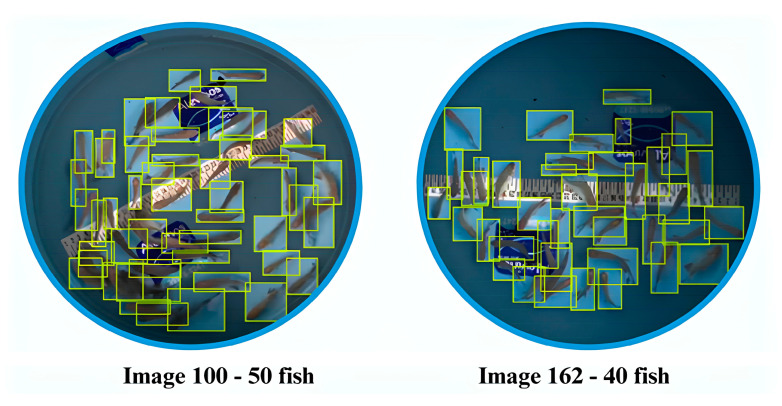
Bounding boxes around the fish, created using Labelimg.

We labeled the digital photographic images using the Roboflow online platform. During the labeling process, we identified each fry in the images by applying bounding boxes (masks) (Figure 3).

A team of 10 trained and experienced individuals labeled the database to minimize errors. We organized the team into two groups. The first group, comprising six undergraduate students from fields such as zootechnics, computer science, and biological sciences, visually analyzed the fish and labeled the specimens with bounding boxes. The second group, consisting of four students from the Postgraduate Programme in Zootechnics, reviewed and corrected the labels after analyzing the images.

The dataset contained images with obstructions and low sharpness, and the water used came from the recirculation system where we cultivated the specimens. We included these images in the training to better evaluate the models’ effectiveness under these conditions and to reflect more practical realities. Before starting the training, we applied preprocessing techniques to the dataset, including resizing, normalizing, and converting the image formats to ensure greater uniformity and quality of the data.

We conducted the training process on a computer equipped with an Intel Core i5-10400 processor at 2.90 GHz (Intel, Santa Clara, CA, EUA), 32 GB of RAM (Dell Inc., Round Rock, TX, EUA), and a 240 GB solid-state drive (SSD) (Kingston Technology, Fountain Valley, CA, EUA). During training, we explored different batch sizes and performed 150 epochs to optimize the model.

### 2.3. Fingerling Detection and Counting

We used an open-source algorithm from GitHub/Google Colab, which Bochkovskiy et al. (2020) developed, to train the fingerling detection model [18,19]. This CNN represents a state-of-the-art real-time object detection and image segmentation model, incorporating advancements in deep learning and computer vision. Bochkovskiy et al. initially trained it on the COCO (Common Objects in Context) dataset, which includes over 330,000 images with annotations for 80 object categories [20]. The designers created the network for various vision AI tasks such as detection, segmentation, pose estimation, tracking, and classification. Researchers widely use the COCO dataset to train and evaluate state-of-the-art models. The extensive pretraining on this dataset enhances the CNN’s speed and accuracy, making it adaptable to a wide range of applications.

We adopted an algorithmic architecture consisting of three fundamental components: the backbone, the neck, and the head. We pretrained the backbone on the ImageNet dataset to extract features from images through convolutions. The neck then connected the feature maps generated by the backbone to the subsequent layers, while the head made predictions of bounding boxes or masks. We applied batch normalization at the end of each convolutional layer to regularize the inputs, thereby enhancing the network’s efficiency and convergence.

**Figure 4:**
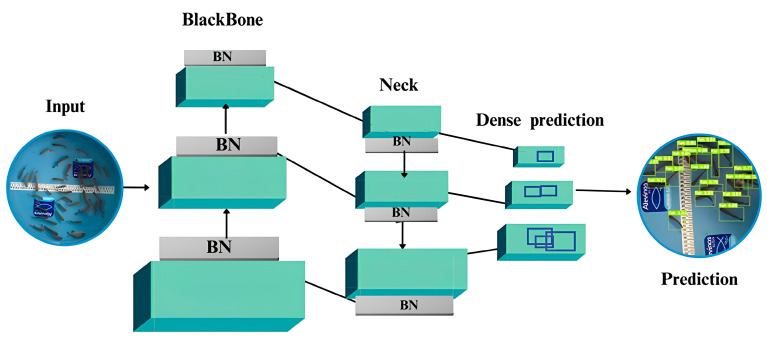
Architecture of the convolutional network used in the study. The blocks in blue represent the network’s numerous convolution layers. The grey blocks correspond to the batch normalization (BN) layers that were applied at the end of the convolution blocks. The network starts with the entrance with dimensions 640 × 640 × 3 (height × width × depth). The first part is the backbone and neck, which includes several layers of convolutions and pooling that reduce the spatial dimension of the input image and extract features. The last part is the dense prediction layers which operate in the dimensions 3 × 3 × 1024, allowing for precise and detailed object detection. The blocks representing the convolution layers are connected by arrows, which represent the operations that alter the spatial dimensions, reducing the width and height of the image, maintaining or increasing the number of channels, while the lines indicate the continuous flow of data, preserving the spatial dimensions between successive layers. Adapted from [20].

The convolutional network exhibited considerable complexity, featuring 415 layers strategically arranged to ensure precise detection of fingerlings at various scales (Figure 4). During training, we explored various convolutional and concatenation operations to enhance progressive feature extraction. We designed the architecture to capture subtle nuances in images, thereby improving the robustness and accuracy of fingerling detection.

Key parameters of the convolutional network are summarized in Table 1.

### 2.4. Evaluation of Metrics

We utilized a total of 692 images for training (69%), allocated 194 images for validation (19%), and designated 114 images for testing (11%). Due to computational constraints, specifically the limited capacity of the computer used for training, we did not apply data augmentation techniques. However, the volume of data collected sufficed to assess the models’ generalizability, and we plan to explore this approach further in future studies. We evaluated the performance of the convolutional network using metrics from the confusion matrix: true positives (TP), false positives (FP), true negatives (TN), and false negatives (FN). TP indicates correctly identified objects, FP represents incorrectly detected objects, TN refers to the number of instances in which the model correctly predicted the negative class; that is, the model did not detect the fish in the image, and in reality, the image did not actually contain the fish, and FN denotes incorrect predictions of the negative class.

**Figure 5:**
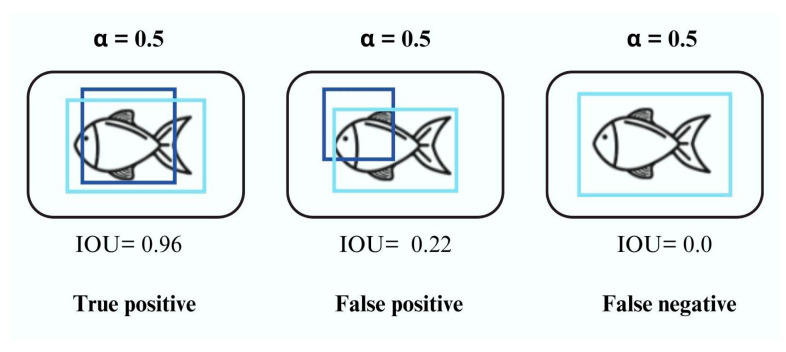
Illustrative scheme of the intersection of the manually defined bounding box (light blue) and that predicted by the network (dark blue). When the overlap (IOU) between them is 50%, the convolutional network identifies the fish as correct.

Fish detection was based on the intersection between manually defined bounding boxes and those predicted by the convolutional network, using a threshold of 50% intersection over union (IoU) (Figure 5). The metrics used to evaluate performance include accuracy (A), precision (P), recall (R), and average precision (mAP@0.5).

Accuracy (A) = (TP + TN)/(TP + TN + FP + FN)

Precision (P) = TP/(TP + FP)

Recall (R) = TP/(TP + FN)

Average precision metric (mAP) = 1/N Ʃ*^N^_i=_*_1_
*A × P*

## 3. Results

In general, we observed that the evaluated metrics—precision, recall, and mAP@0.5—showed lower values with a batch size of 0 (Table 2). As the batch size increased, the metrics tended to improve. A batch size of 20 produced optimal results, achieving a precision of 96.74%, a recall of 95.48%, an mAP@0.5 of 97.08%, and an accuracy of 98%.

**Figure 6:**
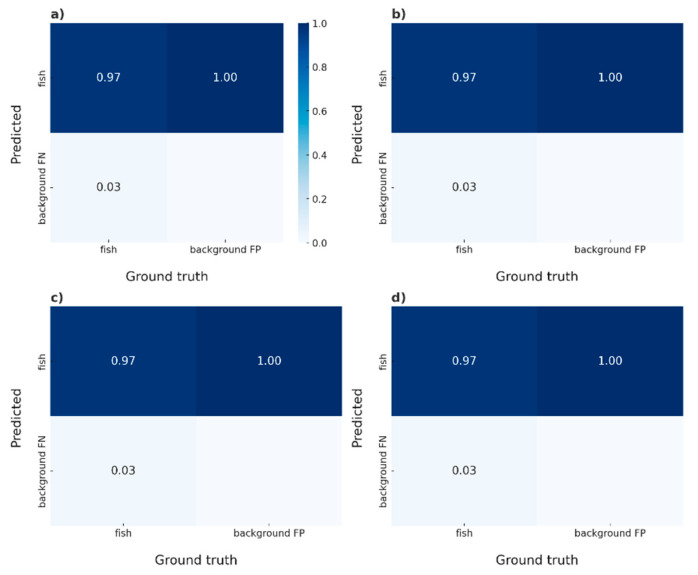
Confusion matrix. (**a**) Without normalization; (**b**) Model with batch size 5; (**c**) Model with batch size 10; and (**d**) Model with batch size 20.

**Figure 7:**
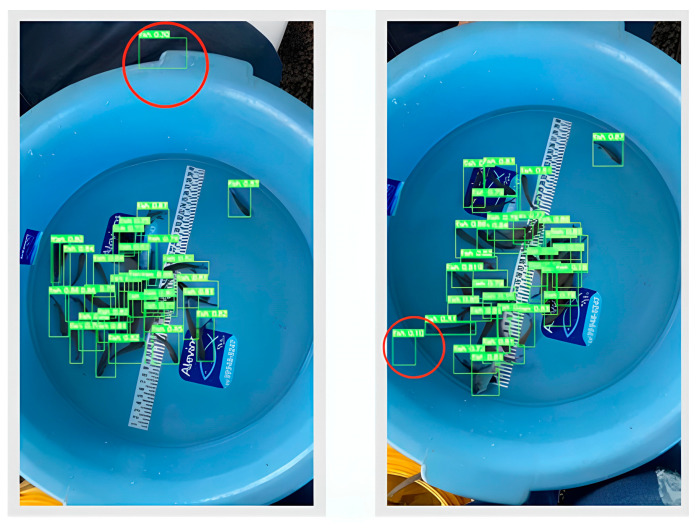
Images with 30 fingerlings detected by the network through mask prediction (light green). Red circles show false positives.

**Figure 8:**
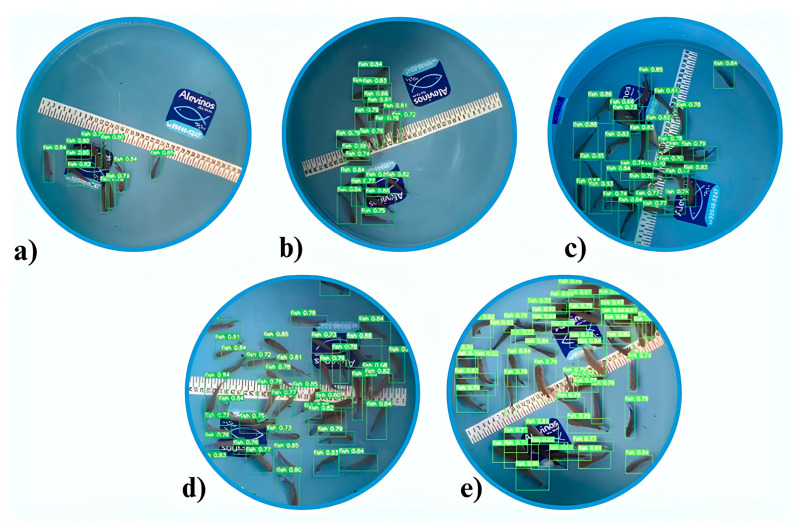
Fry detected by predicting the delimiting bands using the CNN, in light green. (**a**) 10 fry detected; (**b**) 20 fry detected; (**c**) 30 fry detected; (**d**) 40 fry detected; (**e**) 50 fry detected.

Figure 6 shows confusion matrices with rows and columns representing the proportions of false positives, false negatives, true positives, and true negatives. This figure also displays the proportions of predicted values from the trained models. We analyzed the results from these matrices to evaluate the performance and efficiency of the neural network models. In matrices without normalization and with varying batch sizes, we observed that the false negative (FN) rate was higher in batches of 5 and 10 compared to batch 20. Batch 20 demonstrated superior performance in correctly detecting fish, achieving an accuracy of 98% (Figure 7 and Figure 8).

## 4. Discussion

The detection algorithm exhibited satisfactory performance in recognizing and enumerating pirapitinga fingerlings, as evidenced by the mAP@0.5 values and recall exceeding 95%. This validates the accurate predictability of the tested convolutional network. The resulting values were superior to those reported by [6] who found mAP values of 95.4% and a recall of 88% for fish detection in various cultivation systems. However, the tested network encountered challenges in detecting smaller fish.

Although this study did not evaluate the algorithm’s performance in complex environmental settings, such as underwater conditions, it identified other notable limitations. Obstructions and occlusions among the fingerlings, low sharpness in digital images with higher fish density, and variations in the number of fry within the dataset presented significant challenges. These factors can impact the network’s learning and potentially affect accuracy and other metrics [7,21,22]. Despite these challenges, the evaluated model showed partial success in addressing these issues.

The high density of fry can also increase the rate of false negatives (FN) and false positives (FP) in the models. In this study, the confusion matrix revealed no false positives (FP = 0), but all models exhibited some false negative rates. The 50% threshold approach, combined with supervised dataset labelings, kept these rates minimal and did not compromise the effectiveness of detecting fry accurately [23,24].

Regarding the evaluated batch normalization technique, we found that the precision, recall, and mAP values were lower for the model trained without batch normalization compared to those with it. However, the differences between models with various batch sizes were not significant. Larger batch sizes generally led to better performance in the detection algorithm, enhancing metrics such as precision. Nevertheless, increasing batch sizes also raised computational costs [25,26].

A batch size of 10 achieved a precision of 94.23%, while a batch size of 20 yielded a precision of 96.28% [27] for fish detection and classification using an improved CNN. These values closely match those found in this study, confirming that precision increases with batch size [28]. Research on recognizing and counting fingerlings using detection algorithms shows that small batch sizes up to 20 deliver excellent efficiency and performance without significant computational costs [29,30].

Using a batch size of 20 resulted in better stability and convergence during CNN training, with mAP@0.5 percentages slightly exceeding those of other batch sizes, reaching 97.08%. Thus, to achieve optimal performance in detecting pirapitinga fingerlings, we recommend using a batch size of 20 due to computational limitations. Similar studies suggest starting with a batch size of 32 and adjusting it as necessary to avoid unnecessary computational costs while enhancing the accuracy and efficiency of the trained models [29].

High-quality datasets are crucial for achieving excellent results in CNN learning. Creating these datasets involves time-consuming and laborious labeling work [30,31,32]. In this study, the team carefully monitored the labeling process to prevent errors. The dataset included variations to enhance the robustness of the trained models. We used a total of 1000 images, with fingerling densities ranging from 10 to 50 individuals per tank, to build the training set. The images showed slightly turbid water, and we did not use a lighting system to improve visibility. In contrast, Fernandes et al. (2024) used a lighting system to facilitate visualization, achieving metrics above 99% with a much smaller number of images [33,34,35].

Robust data is essential for ensuring that trained models perform efficiently and practically in real-life situations [36]. Although this study collected data from a controlled environment, the observed adverse variations were sufficient to enhance the robustness of the trained networks [26,37]. Therefore, the experimental conditions and models developed for counting pirapitinga fingerlings through images can replace manual methods commonly used in retail businesses, such as sampling with sieves. In such establishments, staff first count the fingerlings before packaging them for transport, often using less turbid water and improved lighting to facilitate visualization.

## 5. Conclusions

In conclusion, the results demonstrated that a batch size of 20 yielded superior precision, accuracy, mAP@0.5, and recall outcomes compared to smaller batch sizes, thereby substantiating its efficacy in the detection of fry.

## Figures and Tables

**Table 1:** Parameters used in the configuration of the convolutional network.

Implementation Details	Parameters
Training	r0 = 0.01 l rf = 0.1, momentum = 0.937 weight_decay = 0.0005 box = 0.05, loss ota = 1 Batch size = 5, 10, 20 Max-epochs = 150 Loss_function = BCE (Binary Cross Entropy) Input_size = 768 × 1024 IOU_thres = 0.45

**Table 2:** Values of the evaluated metrics in the study according to the application of normalization and batch size.

Batch	Precision (%)	Recall (%)	mAp@0.5 (%)	Accuracy (%)
0	96.1	95.38	95.69	97
5	96.37	95.59	96.81	97
10	96.4	95.48	96.28	97
20	96.74	96.01	97.08	98

## Data Availability

Data are contained within the article.
